# Peer Review of “Impact of Weekly Community-Based Dance Training Over 8 Months on Depression and Blood Oxygen Level–Dependent Signals in the Subcallosal Cingulate Gyrus for People With Parkinson Disease: Observational Study”

**DOI:** 10.2196/67813

**Published:** 2024-12-13

**Authors:** Anonymous

**Keywords:** depression, cingulate, GDS, Geriatric Depression Scale, neuroimaging, dancing, Parkinson disease, neurodegenerative, MRI, imaging, neurology, magnetic resonance imaging

*This is the peer-review report for “Impact of Weekly Community-Based Dance Training Over 8 Months on Depression and Blood Oxygen Level–Dependent Signals in the Subcallosal Cingulate Gyrus for People With Parkinson Disease: Observational Study.”*

## Round 1 Review

### General Comments

The paper [1] is interesting in that it proposes to compare depression scores with task functional magnetic resonance imaging (fMRI) measurements in the subcallosal cingulate gyrus (SCG) of people with Parkinson disease (PD) that underwent dance classes over a long time span (around 8 months).

However, it has a major methodological flaw: it correlates depression score changes obtained over 1 day (before and after a dance session) with fMRI signal changes obtained over months. More specifically, it is reported that “A Pearson correlation analysis of the change in GDS data from pre to post (Figure 2B) and the decrease in BOLD signal data showed a strong significant positive correlation…(Figure 2E).” This does not make sense. This correlation should be performed preferably with measures taken at the same time points or, at least, over the same time span.

### Specific Comments

#### Major Comments

1. The correlation between GDS data and blood oxygen level–dependent (BOLD) data should be performed over the same time span.

2. The abstract is misleading since it says that 17 dancers had fMRI scans at 4 time points, but this is not true, since some of those dancers had only 2 or 3 scans. This information (of how many dancers had how many scans) should be in the paper.

3. I am not sure if it is valid to average the BOLD signals of the participants, as was done in Figure 2C. I would like a better justification for this. Also, it should be reported the number of participants that entered the average of each one of the signals.

4. Figure 2, in general, should be better explained in the text.

5. *Introduction*, fifth paragraph: The authors say that “To date, there has been only one fMRI case study with a single participant in which correlations between motor improvements and neural changes were explored.” This is not true—see, for example, [2-5].

6. Still *Introduction*, fifth paragraph: The authors mention the Batson et al [6] study, but it would be relevant for this study to know with which type of and with how many participants this study was conducted.

7. Still *Introduction*, fifth paragraph: The authors mention a recent study but they actually do not say if it was conducted with people with PD (this is implicit because they used Dance for PD, but I believe it should be explicitly stated).

8. *Methods*: “Study population – Neuroimaging sessions over 8-months”—how come the subsample of 10 people with PD has the same demographic characteristics as the total sample of 23 people with PD?

9. *Results*: a “reduction of GDS scores” is mentioned—I assume that GDS score reduction means improvement in depression symptoms? It would be important to mention this somewhere.

10. *Results*: Berg Balance Scale and Timed Up and Go results are mentioned “en passant,” but data are neither shown nor discussed anywhere.

#### Minor comments

11. Figure 2C: What is the x-axis (variable and units)? Also, the y-axis should be relative (and not percentage) change—or were your maximum changes smaller than 1%?

12. Figure 2D: Same comments as for Figure 2C.

13. Figure 2A could be decreased and Figure 2B-E could be increased (I had to set zoom at 400% to be able to see those figures properly).

14. *Introduction*, second paragraph: “with efficacy subject to decay over time”—should it be “subjected” instead?

15. *Methods*, *Procedures*, *Imaging*: I suggest replacing “slice thick” with “voxels.”

16. *Methods*: In the sentence “Following statistical analysis of the BOLD signal, data was conducted in MATLAB,” I believe it should be “data analysis.”

## Abbreviations

BOLD: blood oxygen level–dependent
fMRI: functional magnetic resonance imaging
GDS: Geriatric Depression Scale
PD: Parkinson disease
